# Pediatric-adult split liver transplantation: an ethical imperative and systems-based roadmap for global expansion

**DOI:** 10.3389/frtra.2025.1709770

**Published:** 2026-01-23

**Authors:** Tran Cong Duy Long, Huynh Tran Bao Chau, Dat Tien Le, Thi Tran Nguyen Minh, Bui Cong Minh, Anh Le Thien Diep, Hoang Bao Tran Van, Truc Thi Thu Huynh, Nguyen Tien Huy

**Affiliations:** 1 Department of Hepatobiliary and Pancreatic Surgery, University Medical Center at Ho Chi Minh City, Ho Chi Minh City, Vietnam; 2 Department of Surgery, Faculty of Medicine, University of Medicine and Pharmacy at Ho Chi Minh City, Ho Chi Minh City, Vietnam; 3 College of Health Sciences, VinUniversity, Hanoi, Vietnam; 4 Online Research Club, Nagasaki, Japan; 5 Medical Office, University Medical Center at Ho Chi Minh City, Ho Chi Minh City, Vietnam; 6 Pham Ngoc Thach University of Medicine, Ho Chi Minh City, Vietnam; 7 UF Health North Cardiovascular Center, Jacksonville, FL, United States; 8 Department of Pathology, School of Medicine, Tan Tao University, Long An, Vietnam; 9 Institute of Research and Development, Duy Tan University, Da Nang, Vietnam; 10 School of Medicine and Pharmacy, Duy Tan University, Da Nang, Vietnam; 11 Graduate School of Tropical Medicine and Global Health (TMGH), Nagasaki University, Nagasaki, Japan

**Keywords:** ethical framework, hub-and-spoke model, pediatric organ allocation, split liver transplantation, transplant policy, waitlist mortality

## Abstract

This paradox emphasizes the urgent need to re-engineer transplant systems around pediatric-adult split liver transplantation (SLT). Despite being as effective as whole-liver transplants, SLT remains underutilized due to logistical and policy barriers. We believe that expanding SLT is not only clinically acceptable but a just and effective moral necessity. We propose a five-point action plan, including centralized splitting hubs, NMP-enabled transport, and global pilot programs, to transform SLT from an exception into standard practice.

## Introduction

Even though there were 27 children died on the U.S. liver waitlist in 2023, yet more than half of potentially split-able livers were transplanted whole into adults (1). From a global perspective, the increasing recipient pool prompts rapid action to increase the number of available grafts due to the rising prevalence of alcoholic liver disease, non-alcoholic steatohepatitis (NAFLD) and Hepatocellular carcinoma (HCC) as a result of a sedentary lifestyle (2, 3). In many Asian countries, especially Japan which pioneered and relies heavily on Living donor liver transplantation (LDLT), the main problem faced by their healthcare system is a shrinking pool of potential donors due to low birth rates and declining population (4). And due to the poor prognosis nature of most paediatric acute liver failure (PALF) cases, the living donors’ graft pool could not minimise the need to wait for a perfectly size-matched whole liver, resulting in most PALF cases being excluded from the waitlist (5).

**Figure 1:**
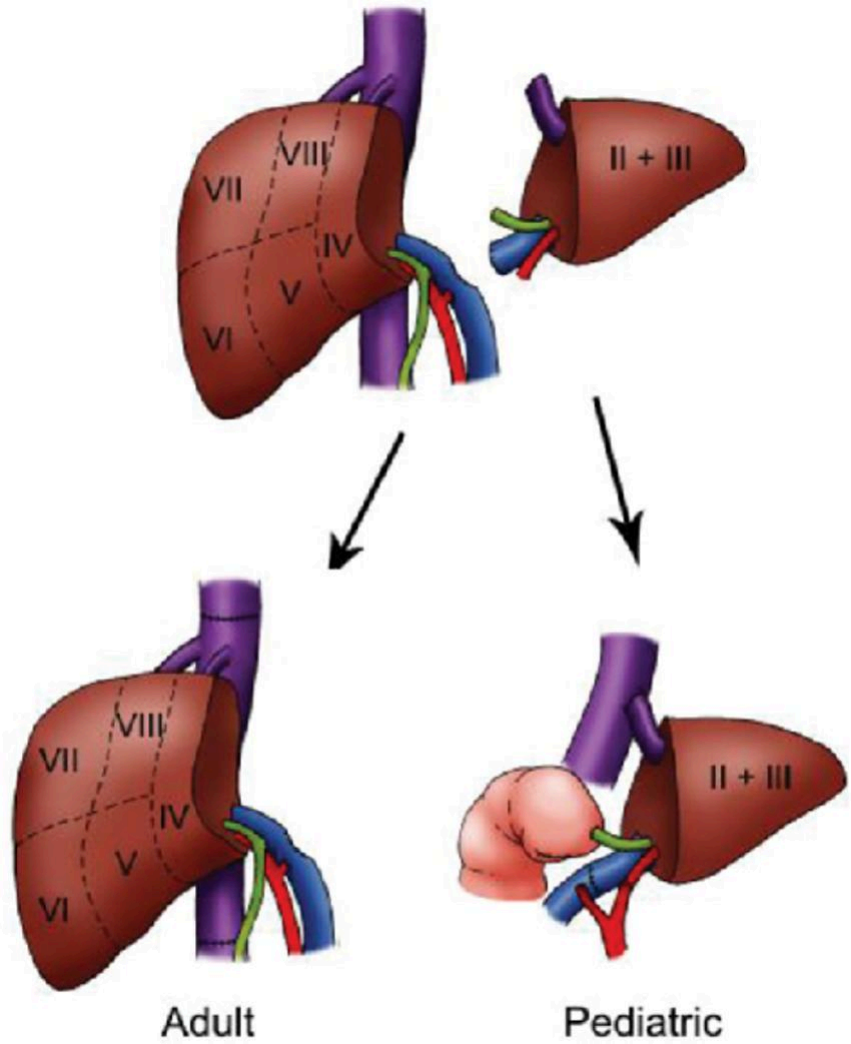
Examples of surgical techniques for orthotopic liver transplantation. Adapted from Wiederkehr J. et al. (7).

Liver transplantation is a common technique in which the healthy liver sample is transferred from a donor to a recipient with severe hepatic conditions through a liver waitlist system. Split liver transplantation (SLT) utilises only one of the liver's functioning lobes to be transplanted with the hope that the other lobe would regenerate inside the recipient's body. In pediatric–adult split liver transplantation, one donor liver is split into a left lateral segment for a pediatric recipient and an extended right graft for an adult, typically a small-statured recipient (6) (Figure 1).

This technique has been shown to be feasible and can expand the donor pool, particularly in settings with significant pediatric organ shortages (6, 8). Compared to LDLT, SLT provides recipients with shorter waitlist times, thereby reducing pre-transplant mortality (9). Although SLT is associated with higher short-term graft failure rates, both graft and patient survival remain comparable to those observed with LDLT (9). However, SLT remains underutilized in many countries due to logistical, institutional, and policy-related barriers; this could be linked to an alarming mortality rate of young recipients who did not receive the graft in time (6). Therefore, this article supports the SLT approach to become a standard procedure in modern healthcare.

## Current status of organ transplantation in Vietnam

Vietnam's transplant program has expanded significantly since the first kidney transplant in 1992. By 2025, the Ministry of Health reported nearly 10,000 transplants, including 8,900 kidney, 750 liver, 126 heart, and 13 lung procedures (10). More than 20 hospitals are now certified to perform transplantation, with Viet Đức Friendship Hospital (Hanoi) and University Medical Center Ho Chi Minh City serving as national leaders. Academic case series confirm technical sophistication: for example, pediatric liver transplantation for Budd–Chiari syndrome has achieved outcomes comparable to international standards (11).

### Donor sources and persistent challenges

Despite technical success, Vietnam faces severe donor scarcity. Over 95% of grafts are from living donors, reflecting cultural reluctance and logistical barriers to deceased donation (12). Although donations from brain dead patients have increased in recent years, Vietnam remains among the lowest globally in deceased donor rates (12).

### Data collection and reporting

Unlike the United States’ OPTN registry, Vietnam does not maintain a centralized, publicly accessible waitlist database. Instead, data are reported through Ministry of Health announcements, hospital press releases, and academic publications (10). This fragmented reporting limits transparency and hinders equitable allocation.

### Allocation systems

The Model for End-Stage Liver Disease (MELD) score is a validated prognostic system originally developed to predict short-term mortality in patients undergoing transjugular intrahepatic portosystemic shunt (TIPS) procedures (13). It was later widely adopted in hepatology and liver transplantation because of its objectivity, reproducibility, and strong predictive accuracy for 3-month mortality among patients with advanced liver disease (13). In several countries, particularly those with centralized transplant waitlists such as the United States and parts of Europe, MELD-based prioritization forms the foundation of liver allocation policies, ensuring that donor livers are allocated according to medical urgency (14). However, Vietnam does not maintain a unified national transplant waitlist or an officially published liver allocation policy. Available reports focus primarily on donation activity and transplant outcomes rather than allocation frameworks, and there is no published evidence that MELD is formally used as the basis for liver allocation nationally. Instead, patient assessment and transplant decision-making occur at the level of individual transplant centers, leading to heterogeneous practices and limited transparency across institutions.

## The ethical and clinical imperative

Because children require size-appropriate grafts, techniques such as SLT remain crucial in enhancing access for pediatric candidates. Data in the US between 2010 and 2015 shows that only 6.3% of deceased-donor livers met criteria for potential splitting, yet just 3.8% were split; 96% served only a single adult recipient, leaving opportunities for pediatric benefit unrealized (15). Globally, the underused split graft persisted between 2011 and 2013; just 26.5% of pediatric liver transplants utilized partial or split grafts, while nearly two-thirds still relied on whole livers (16). The consequences are profound. Approximately one in ten infants and one in twenty older children in the U.S. die before receiving a liver transplant (15).

This reality underscores both an ethical and clinical mandate for broader application of split-liver transplantation (SLT). When life-saving resources are scarce, ethical analyses emphasize obligations to allocate organs in ways that maximize benefit and promote fairness (17). Three well-established concepts in pediatric ethics are directly relevant. First, the fair-innings argument asserts that younger individuals, who have not yet had the chance to experience a normal lifespan, have a stronger claim to life-extending resources (17, 18). Second, prudential lifespan accounts argue that society should distribute scarce resources in a way that enables individuals to achieve a complete and continuous life narrative, thereby supporting prioritization of those at the beginning of life (19, 20). Third, principles of justice and equity require attention to systematic disadvantages faced by children, whose small size and developmental vulnerability constrain access to adult-oriented organ allocation systems (21, 22).

These obligations are echoed by policy bodies: the OPTN Ethics Committee affirms that splitting livers enhances efficiency without disadvantaging pediatric recipients (23). Similarly, the International Pediatric Transplant Association (IPTA) advocates pediatric prioritization and recognizes SLT as a vital means of equitable organ distribution (19).

Importantly, clinical outcome data reinforced this ethical approach. A study reveals that SLT pediatric recipients have a 95% one-year survival rate and 90% graft survival rate (16). Taken together, ethical principles, policies, and strong clinical evidence make a compelling case: SLT is not only technically and clinically viable, but also an ethical imperative to address preventable pediatric deaths.

## Adult-adult split liver transplantation

Adult-adult SLT involves dissecting the donor liver graft according to the Cantlie line, reserving both the left and right hemilivers, containing segments I-IV and segments V-VIII, respectively, for adult recipients (24). This differs from pediatric-adult SLT as pediatric recipients only require segment II-III from the left hemiliver.

Adult–adult split liver transplantation (SLT) has become a viable strategy to increase the donor pool for adult recipients, although pediatric–adult SLT remains the primary ethical and clinical priority. Improved graft selection, surgical technique, and perioperative management have significantly allayed early worries about vascular insufficiency, biliary complications, and small-for-size syndrome. According to recent multicenter analyses, 1-year graft survival rates are between 85 and 90 percent, and patient survival is close to 90–95 percent. These results are now on par with whole-liver transplantation (25–27). The key advantages of adult–adult SLT include: expanding organ availability by providing two adult recipients from a single donor (28); reducing wait-list mortality, especially in regions with severe donor scarcity and enabling equitable allocation of large, high-quality livers that might otherwise go underutilized.

Still, several difficulties remain. The technical complexity of parenchymal transection and biliary reconstruction enhances the risk of bile leakage or early graft dysfunction after surgery, and adult-adult SLT necessitates careful donor-recipient matching to prevent small-for-size grafts (26, 27). Furthermore, not all donor livers can be split anatomically, and the best results are obtained in busy facilities with standardized procedures and multidisciplinary knowledge. Despite these drawbacks, there is mounting evidence from around the world that adult-to-adult SLT is a safe and effective procedure. This supports its role as a common transplant option that works in tandem with pediatric-to-adult SLT to achieve a fair and life-saving use of limited organs (25, 28).

## Current barriers to expansion

Although split-liver transplantation (SLT) offers a promising avenue for substantially augmenting the available donor liver pool, its global implementation is still restricted. A primary impediment stems from the intricate technical demands of the procedure, which necessitates surgeons possessing expertise in graft partitioning, vascular and biliary reconstruction, and the capacity for precise intraoperative judgment. Consequently, SLT is predominantly performed within high-volume transplant programs that have cultivated established multidisciplinary proficiency, rather than being readily available throughout transplant networks (8). Furthermore, disparities in geographic access exacerbate the problem. Smaller or resource-limited centers frequently lack the specialized personnel, perioperative infrastructure, and coordinated logistical support essential for the safe execution of SLT. Consequently, potentially suitable grafts for splitting may be transplanted in their entirety or underutilized, primarily due to the absence of appropriate pathways, policy incentives, or inter-center coordination mechanisms (15, 29). These persistent obstacles highlight the need for structured training programs, standardized protocols, and resource distribution strategies that incorporate SLT into regular medical practice. Without intentional planning at the system level, SLT will continue to be a specialized procedure, rather than a scalable solution to the shortage of donors and the preventable mortality rates on pediatric and adult waiting lists.

## A proposed five-point action plan

It is imperative, from the imminent organ shortages and long waiting time, to develop nationwide or worldwide guidelines upon SLT and capable surgical centers with adequate expertise in SLT techniques. In China, a consensus states the agreement upon critical procedures such as donor and recipient selection, graft evaluation, surgical techniques including splitting forms and vascular or biliary tracts allocation, and perioperative management (30). And only with experienced transplant centers do the outcomes of SLT patients become matchable with those of traditional liver transplant because of standardised donor graph evaluation, logistics, and postoperative management (8).

Secondly, an allocation framework is needed to provide the necessary logistics for any successful surgery. In Italy, the Mandatory Split policy states that all deceased patients aged 18–50 without baseline liver diseases and stable hemodynamics must be offered to pediatric surgical centres for transplantation, unless an adult patient is on a whole-liver transplant waiting list, therefore helping the country to achieve a staggering pediatric SLT graph receipt rate of 65.8% as well as dropping wait-list time and mortality (31). A similar policy should be created to combine SLT into the existing transplant allocation framework to make SLT a mandatory medical consideration, as well as prioritising pediatric patients in graft distribution.

Despite its efficacies, SLT has many downsides that hamper it from becoming effective, such as long Cold Ischemia Time (CIT) and logistical difficulties connecting faraway patients. The Hub-and-Spoke model proposes a “Hub” or high-volume transplant centre capable of splitting a liver, which distributes to a “Spoke” or low-volume transplant facility capable of normal transplantation. A normothermic machine perfusion system will help deliver the graft while maintaining live tissue at its physiologic condition, reducing tissue damage (32). Allocation is done by Artificial Intelligence, by successfully matching grafts with appropriate recipients, it significantly reduces any damage to the liver and saves precious time (33).

There are many models of a national registry that receive comprehensive transplant data. On the national level, the China Liver Transplant Registry was contributed to by South China Split Liver Transplantation Alliance, a network of 25 hospitals conducted a total of over 300 SLT cases, in an effort to further standardise SLT across the country (34). To continue allocation efficiency while promoting transparency and equity, more data upon donor eligibility, splitting logistics, center capability, waitlist mortality, and patient or donor outcomes should be collected.

A pilot program should be conducted, although not yet on a global scale, on a national or multinational level. The Johannesburg Pediatric Program was initiated by Donald Gordon Medical Centre since 2005, and it has solved the need for liver transplant of 270 pediatric patients through methods like SLT (35). They have since then received funding from both private and public sectors, accepting public hospital patients, and publishing annual data without the presence of the South African national registry, which are great initiatives for any future pilot program (36).

## Compare SLT with living donor transplantation

Although both methods utilize partial liver grafts, pediatric-adult SLT uses deceased donor livers while Living donor liver transplantation (LDLT) uses healthy living donor organs. SLT has provided unprecedented mortality rates for pediatric patients. LDLT also significantly improves mortality of recipients and has been proven to have better post-surgical prognosis regarding early vascular complications, hepatic artery thrombosis, and biliary complications than SLT (37). Both techniques benefit from recent surgical advancements such as *in situ* and *ex vivo* splitting and machine perfusion for SLT, or non-invasive laparoscopic surgery in living donors (38). However, LDLT still places the donor at a significant risk for severe complications such as sepsis and stomach gas gangrene leading to estimated mortality rate from 0.15% to 0.4% which is five times greater than that of kidney transplant; an estimated 31% post-surgical complication and increased socioeconomic burdens (39). LDLT procedure numbers are also plateauing from 2001 as a result of donor's fear of transplant complications and changing organ allocation (39, 40).

## Conclusion

In conclusion, pediatric–adult SLT is more than a technical innovation; it is a necessary response to persistent inequities in organ allocation and the preventable deaths of vulnerable children. As global systems vary widely, from MELD-based prioritization in some countries to the absence of unified waitlists in others, SLT offers a practical and ethical solution to expand access across diverse settings. We urge the transplant community, ethicists, policymakers, and clinicians to recognize SLT as both a moral responsibility and a system-level priority. With aligned policies, coordinated logistics, and transparent data reporting, SLT can shift from an exceptional procedure to a routine standard of care, ensuring that every donor liver achieves its fullest life-saving potential.

## Data Availability

The original contributions presented in the study are included in the article/Supplementary Material, further inquiries can be directed to the corresponding author.
